# Best practice guidelines for blunt cerebrovascular injury (BCVI)

**DOI:** 10.1186/s13049-018-0559-1

**Published:** 2018-10-29

**Authors:** Tor Brommeland, Eirik Helseth, Mads Aarhus, Kent Gøran Moen, Stig Dyrskog, Bo Bergholt, Zandra Olivecrona, Elisabeth Jeppesen

**Affiliations:** 1 Department of Neurosurgery, Oslo University Hospital Ullevål, Kirkeveien 166, 0450 Oslo, Norway; 2 Faculty of Medicine, University of Oslo, Problemveien 7, 0315 Oslo, Norway; 3 Department of Neuromedicine and Movement Science, Norwegian University of Science and Technology, Trondheim, Norway; 4 Department of Medical Imaging, Nord-Trondelag Health Trust, Levanger, Norway; 5 Department of Neurointensive care, Aarhus University Hospital, Nørrebrogade 44, 8000 Aarhus, C, Denmark; 6 Department of Neurosurgery, Aarhus University Hospital, Nørrebrogade 44, 8000 Aarhus, C, Denmark; 7 Department of Anestesia and Intensive care, Section for Neurosurgery, Faculty of Health and Medicine, Department for Medical Sciences, Södre Grev Rosengatan, 70185 Örebro, Sweden; 8 National Trauma Registry, Department of Research and Development, Division of Orthopedics, Oslo University Hospital, NO-0424 Oslo, Norway

**Keywords:** Vascular injury, CT angiography, Screening, Trauma, Guidelines

## Abstract

Blunt cerebrovascular injury (BCVI) is a non-penetrating injury to the carotid and/or vertebral artery that may cause stroke in trauma patients. Historically BCVI has been considered rare but more recent publications indicate an overall incidence of 1–2% in the in-hospital trauma population and as high as 9% in patients with severe head injury. The indications for screening, treatment and follow-up of these patients have been controversial for years with few clear recommendations. In an attempt to provide a clinically oriented guideline for the handling of BCVI patients a working committee was created. The current guideline is the end result of this committees work. It is based on a systematic literature search and critical review of all available publications in addition to a standardized consensus process. We recommend using the expanded Denver screening criteria and CT angiography (CTA) for the detection of BCVI. Early antithrombotic treatment should be commenced as soon as considered safe and continued for at least 3 months. A CTA at 7 days to confirm or discard the diagnosis as well as a final imaging control at 3 months should be performed.

**Electronic supplementary material:**

The online version of this article (10.1186/s13049-018-0559-1) contains supplementary material, which is available to authorized users.

## Background

Blunt cerebrovascular injury (BCVI) is a non-penetrating injury to the carotid and/or vertebral arteries. The pathological mechanism is thought to be stretching or impingement of the vessel walls as the head and neck is forcefully moved in flexion, extension or rotation. This causes intimal tear with exposure of subintimal layers to the blood flow and consequently thrombus formation, wall hematoma and even lumen occlusion. In some instances this process develops into a pseudoaneurysm [1]. BCVI has been given considerable attention in the literature for the past 30 years [2–5]. It was earlier considered to be a very rare injury but recent publications show an incidence of 1–2% in the in-hospital trauma population and 9% in patients with severe head injury [6, 7]. BCVI is clearly associated with severe facial injuries and fractures of the skull base and cervical spine [8–15]. Thrombus formation at the site of an intimal tear may occlude the vessel or shed an emboli to a more peripheral brain artery, both processes resulting in a stroke. The true incidence of such an ischemic event due to BCVI is largely unknown, but reported in the range of 1–26% in recent literature [6, 16–19]. There seem to be a higher risk of ischemic events with injury to the carotid than the vertebral artery [19, 20]. BCVI is an independent predictor for poor outcome with higher morbidity and mortality rates in trauma patients with this injury, reported as high as 25–50% for those suffering a stroke [5, 19].Unfortunately, a substantial number of patients with this injury arrive at the hospital with a stroke in progress [6]. Treating the remaining asymptomatic patients with BCVI in order to avoid an ischemic insult is controversial [17, 21, 22]. There have been numerous publications on the topic including systematic reviews. However, for the clinician working with trauma patients, the literature gives few specific recommendations that aid in the daily handling of this injury.

In this systematic review we raise the following clinical questions: 1. What part of the trauma population should be screened for BCVI? 2. Which radiological method should be applied for screening? 3. How should BCVI be treated? 4. How should patients with BCVI be handled over time? This is an attempt to provide «best practice» recommendations based on a systematic literature search, careful review of all available publications and methodical evaluation of the evidence.

## Methods

An interdisciplinary working group consisting of five neurosurgeons (TB, ZO, MA, EH and BB), one anesthesiologists (SD) and one radiologist (KGM) was recruited through the Scandinavian Neurotrauma Committee (SNC-www.neurotrauma.nu). In addition, a research methodologist aided in the systematic evidence work (EJ). Key clinical questions were formulated according to the PICO model (Population, Intervention, Comparison and Outcome) (Table 1). All searches were limited to Scandinavian and English language sources. Initial searches were performed to identify existing guidelines in national databanks in each Scandinavian country (Prosedyrer i Nasjonalt nettverk for fagprosedyrer (NO), Nasjonale retningslinjer for Helsedirektoratet (NO), Socialstyrelsen, Nationella riktlinjer (SE) and Sundhedsstyrelsen (DK)) as well as UpToDate, National Guideline Clearinghouse, NICE guidelines and BMJ Best Practice. Systematic reviews where then inquired for through McMaster PLUS, Epistomonikos and The Cochrane Library. Systematic searches for primary articles with the aid of a research librarian were performed on February 11, 2016 with the mesh terms «carotid artery» and/or «vertebral artery», «injury» and/or «trauma» (Additional file 1). Primary studies were found in OVID medline, PubMed and Embase. The search was automated for OVID Medline and PubMed so that the final database included primary studies and systematic reviews through March 31, 2018. Titles were screened and abstracts read for all articles in English dealing with BCVI. Full text primary publications were read, critically reviewed and included if relevant and presenting own patient material. Case presentations with less than five patients were excluded. Grading the quality of evidence and strength of recommendations were conducted according to the GRADE approach [23]. Evidence was rated as high, moderate, low or very low. Strength of recommendations was either strong or conditional. The final guidelines were evaluated in Oslo June 4, 2018 with collaborating medical doctors in a Delphi process utilizing the AGREE II instrument [24].

**Table 1:** The PICO model: Population, Intervention, Comparison and Outcome

Clinical question	P	I	C	O
What part of the trauma population should be screened for BCVI?	In-hospital trauma population	Clinical critera	Various screening criteria	Indications for radiological investigation
Which radiological method should be applied for screening?	Selected trauma population	Angiogram	CTA versus DSA	Vessel injury
How should BCVI be treated?	Trauma patients with vessel injury on angiogram	Medical or interventional treatment	Medical versus interventional versus no treatment	Stroke
How should patients with BCVI be handled over time?	Trauma patients with vessel injury on angiogram	Follow-up controls	Life long versus period of treatment	Stroke

*CTA* CT angiography, *DSA* digital subtraction angiography

## Results

**Fig. 1:**
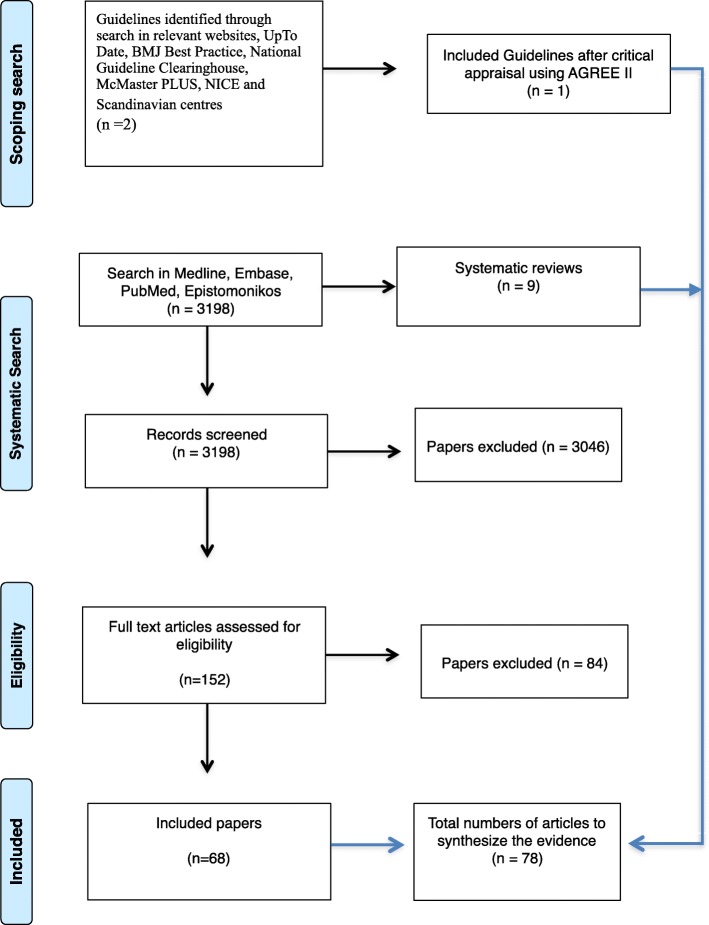
PRISMA flow diagram for selection of included studies

Our results and recommendations are summarized in Table 2. All recommendations apply to both adults and children. No recommendations in the national databanks were found. Two existing guidelines were found of which one was included (UpToDate) and the other found to be equivalent of an already included systematic review [25, 26] A total of 3198 titles were discovered through the remaining searches. Of these, nine systematic reviews were added to our database of which the work by Bromberg et al. was considered to be of particularly high methodological quality with clear clinical recommendations [26–34]. However, the search performed by Bromberg et al. ended in 2005 and all eligible papers published after this date were included. This resulted in a total of 78 articles composing our literature database for synthesis of the guidelines (Fig. 1 and Additional file 2). In general, the scientific evidence was found to be of low or moderate levels. Despite this, strength of recommendations were in some instances set as «strong» as the potential benefits were considered to outweigh possible risk factors. This guideline is an update of existing recommendations with a focus on advising the clinician in handling the trauma patient with BCVI.

**Table 2:** Overview of clinical recommendations, strength, level of evidence and scientific rationale

Clinical question	Recommendation	Strength of recommendation	Level of evidence	Rationale (Benefits and harms)
What part of the trauma population should be screened for BCVI?	Apply expanded Denver screening criteria	Strong	Low	A documented screening tool ensures focus on the condition. Possible danger of overtriage and unnecessary use of imaging.
Which radiological method should be applied for screening?	CTA has acceptable specificity and sensitivity. DSA remains gold standard	Strong	Moderate	DSA is time consuming, invasive with potential complications and often not available 24–7. CTA is fast and available with lower complication risk. CTA has higher radiation exposure with a risk of false positive findings.
How should BCVI be treated?	Early treatment with either LMWH or AP medication	Strong	Low	Uncertainty of treatment effect. Studies show that early treatment is safe. Risk is worsening of existing hemorrhage.
	Continue treatment with LMWH or AP for at least 3 months	Strong	Low	Long term AP treatment is generally safe, but may cause side effects such as peptic ulcer.
	Pseudoaneurysm or high-grade vessel injury may be considered for endovascular treatment	Conditional	Low	May prevent new or recurrent stroke, but uncertainty of treatment effect or stent patency. Double platelet-inhibitors increases risk of hemorrhage in trauma patients.
How should patients with BCVI be handled over time?	Perform re-imaging at 7 days and 3 months.	Conditional	Low	Repeat imaging can confirm or discard the diagnosis of BCVI. Risk is radiation exposure.

*BCVI* blunt cerebrovascular injury, *CTA* CT angiography, *DSA* digital subtraction angiography, *LMWH* low molecular weight heparin, *AP* anti-platelet

### Clinical question 1: What part of the trauma population should be screened for BCVI?

#### Recommendation

All hospitals dealing with a general trauma population should have a systematic and evaluated set of screening criteria in order to assess patients for BCVI. Of existing screening tools we recommend the expanded Denver screening criteria for both adults and children (Table 3). These criteria apply to trauma patients with signs or symptoms of BCVI or a high-energy trauma mechanism with one or more of the risk factors listed in Table 3.

**Table 3:** The expanded Denver screening criteria for BCVI. CT angiography is indicated if one or more of the criteria are present

Signs/symptoms of BCVI	
Arterial hemorrhage from neck/nose/mouth	
Cervical bruit in patients < 50 years	
Expanding cervical hematoma	
Focal neurological deficit	
Neurological exam incongruous with head CT findings	
Stroke on secondary CT scan	
Risk factors for BVCI *(High-energy transfer mechanism with):*	
Le Fort II or III	
Mandible fracture	
Complex skull fracture/basilar skull fracture/occipital condyle fracture	
Severe traumatic brain injury (TBI) with GCS < 6	
Cervical spine fracture, subluxation or ligamentous injury at any level	
Near hanging with anoxic brain injury	
Seat belt abrasion with significant swelling, pain or altered mental status	
TBI with thoracic injury	
Scalp degloving	
Thoracic vascular injury	
Blunt cardiac rupture	
Upper rib fracture	

From Geddes et al.: Expanded screening criteria for blunt cerebrovascular injury: A bigger impact than anticipated (Geddes et al., 2016)

Strength of recommendations: Strong.

Level of evidence: Low.

#### Evidence and rationale

In 1998 Biffl et al. demonstrated that screening could identify asymptomatic patients with BCVI [35]. Other groups have presented similar works [36, 37]. It has been estimated that even with such screening criteria as many as 20% of patients with BCVI may go undetected due to the heterogeneity inherent to this population and the imperfectness of screening tools [37, 38]. However, studies have shown that focus on this condition in a trauma setting and implementation of standardized handling increases the detection rate and may even decrease stroke rate through earlier imaging and treatment [19, 34, 39–45]. Even though no direct comparative studies examining the diagnostic yield of each screening tool exist, the work by Biffl et al. is probably the most extensive and best evaluated [38, 40, 43, 46]. Later known as the Denver screening criteria this clinical tool focus on signs and symptoms of BCVI as well as specific risk factors associated with a high-energy transfer mechanism [38]. It incorporates all indicators also applied by the so-called Memphis screening tool [47, 48]. In the most recent version of the Denver criteria several new risk factors for BCVI has been identified and the expanded screening criteria now includes all patients with cervical spine fractures [40]. This development is supported by others including a meta-analysis demonstrating a 5-times greater likelihood of BCVI in trauma patients with cervical spine fractures compared to those without [49]. In our Delphi consensus meeting such a liberal screening policy was discussed and met with criticism for risk of over-triage and unnecessary radiation exposure. There is a clear need for further studies investigating the diagnostic yield of CTA with the expanded Denver screening criteria and whether they alter patient management or reduce the stroke rate in trauma patients.

Several authors have demonstrated that BCVI do occur in traumatized children and that the incidence may be as high as in adults [28, 50, 51]. An attempt to create a pediatric screening tool in order to minimize the use of radiation exposure from CT angiography (CTA) has also been made [52, 53]. However, in a retrospective study on 7440 pediatric trauma admissions this so called Utah-score failed to accomplish the same detection rate as the Denver screening criteria and other authors have recommended that pediatric trauma patients are managed as adults with respect to imaging for BCVI [54, 55]. In a recent report by Herbert et al. the Utah score was further developed by adding an analysis of the mechanism of injury defining high-risk groups in pediatric trauma patients [56]. Though the authors claim that this adds an improved detection rate compared to both the Denver and Memphis screening tools the difference is in our opinion not clinically relevant.

### Clinical question 2: Which radiological method should be applied for screening?

#### Recommendation

A CT angiography (CTA) of the precerebral carotid and vertebral arteries extending through the base of the skull and including the circle of Willis should be performed in those patients meeting one or more of the Denver screening criteria. A minimum of 16-channel CT technology should be applied. If discovered, vessel injury should be classified according to the Biffl injury grading scale (Table 4).

**Table 4:** The Biffl injury grading scale for BCVI

Biffl injury grade	Angiograhic characteristics
I	Luminal irregularity or dissection with < 25% luminal narrowing
II	Dissection or intramural hematoma with ≥25% luminal narrowing
III	Pseudoaneurysm
IV	Occlusion
V	Transection with free extravasation

From Biffl et al.: Blunt carotid arterial injuries: implications of a new grading scale (Biffl et al. 1999)

Strength of recommendations: Strong.

Level of evidence: Moderate.

#### Evidence and rationale

Digital subtraction angiography (DSA) still remains the gold standard in detecting BCVI [32, 37, 57, 58]. However, this technique is time consuming and not offered by all institutions as a full-time, 7-days a week service. DSA carries a higher risk of procedure-related complications than CTA such as stroke, pseudoaneurysm and hematoma at the site of vessel puncture [59, 60]. In a meta-analysis of CTA versus DSA in BCVI diagnosis it was demonstrated a great variation in published sensitivity and specificity for CTA [33]. The demonstrated pooled sensitivity and specificity for BCVI detection with CTA were 66% and 97%, respectively. This was possibly due to variations in diagnostic threshold and experience across the respective trauma institutions. There seem to be a clear correlation between improved CT technology and diagnostic accuracy: Modern CT scanners with 16-, 32- and 64 -channel technology demonstrate higher sensitivity and specificity with increasing number of slices per rotation [58, 59, 61–63]. In a cost-effectiveness study by Malhotra et al. CTA was found to be superior to DSA in patients selected for imaging based on the Denver screening criteria [42]. Eastman and co-workers significantly reduced time from injury to diagnosis of BCVI from 31.2 h to 2.65 h when converting from DSA to CTA. The stroke rate was also significantly reduced from 15.2 to 3.8%. As medical therapy before and after CTA implementation remained the same, this reduction in ischemic events have been attributed to earlier start of treatment [45].

There seem to be a consistent finding that CTA may detect almost all clinically significant BCVIs as very few strokes have been observed in trauma patients with a negative CTA [57, 59, 61, 62, 64, 65]. DSA is in our opinion impractical as a primary imaging tool as the expanded clinical screening criteria now make more patients eligible for imaging.

Even though MRI technology and availability have greatly improved over the past years few studies using MR angiography for BCVI have been performed. Though the technique may offer comparable sensitivity and specificity as that of CTA it remains impractical and time consuming as a screening tool for the multi-traumatized patient. Ultrasound is observer-dependent and visualizing the entire vertebral artery is challenging [66].

When BCVI is detected, we recommend the use of a grading scale for prognostication and comparison with repeated imaging (Table 4). The so-called Biffl injury grading scale has been extensively used and demonstrate a good intra -and interrater reliability [67].

### Clinical question 3: How should BCVI be treated?

#### Recommendation 1

Antithrombotic therapy should be initiated as soon as considered safe. Early antithrombotic therapy may be commenced even in the setting of severe head injury or other solid organ injury.

Strength of recommendation: Strong.

Level of evidence: Low.

#### Recommendation 2

**Fig. 2:**
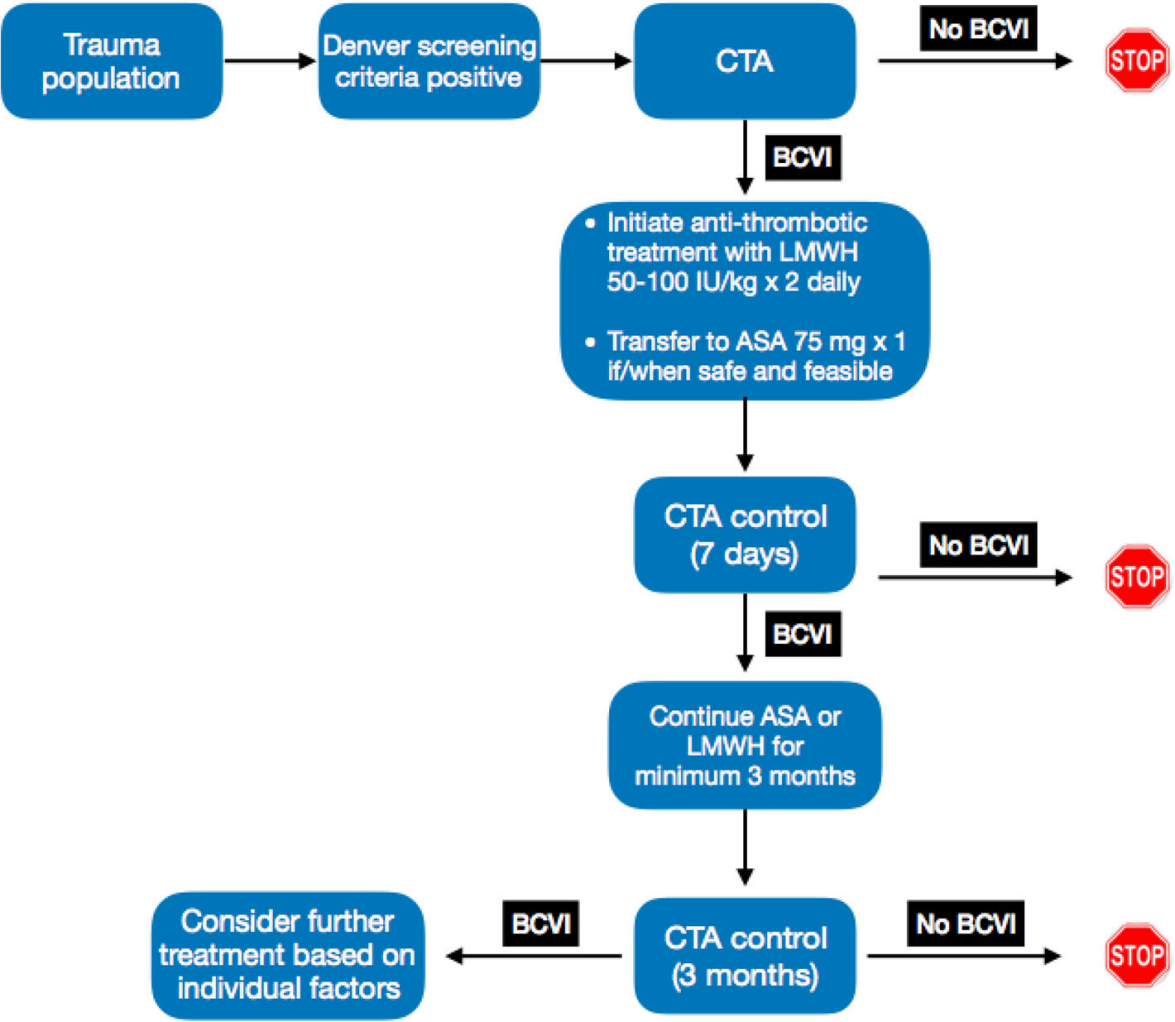
Flow-diagram summarizing the current guidelines for screening, treatment and followup of patients with BCVI

Treatment may consist of anti-platelet or anti-coagulation therapy. We recommend initiation of low-molecular weight heparin (LMWH) in antithrombotic doses within 24–48 h of the diagnosis followed by transfer to oral acetyl salicylic acid (ASA) 75 mg daily when appropriate. In pediatric cases, 3–5 mg/kg of ASA is recommended. The treatment should be continued for at least 3 months (Fig. 2).

Strength of recommendation: Strong.

Level of evidence: Low.

#### Recommendation 3

For pseudoaneurysms progressing in size or severe luminal stenosis producing thrombotic and/or ischemic events a neurointerventionist with endovascular expertise should be consulted.

Strength of recommendation: Conditional.

Level of evidence: Low.

#### Evidence and rationale

Historically, BCVI was often retrospectively detected in the setting of stroke in a trauma patient. With the introduction of screening criteria and non-invasive diagnostics the incidence of asymptomatic BCVI has increased [22]. This has made medical intervention possible in hopes that an ischemic event may be avoided. However, treatment effects with respect to stroke rates vary between studies and the results are conflicting. [17–19, 21, 22, 43, 68, 69]. Possible benefits of antithrombotic treatment must be weighed against any potential risks such as worsening of intracranial hemorrhage or existing hematomas in other solid organs as these patients often have multiple injuries. However, recent studies on BCVI and concomitant intracranial, intraspinal or solid organ injuries have shown no difference in hemorrhagic worsening between treated or untreated patients [7, 43, 68, 70, 71].

Based on the existing literature it is difficult to clearly decide whether treatment of patients with BCVI make a clinically significant difference. This is due to the retrospective nature and small sample sizes of the publications, lack of randomized studies and poorly defined criteria for diagnosing a stroke in this heterogeneous trauma population. On the other hand, knowing that stroke may appear as early as minutes after the injury and as late as days and even weeks it seems rational to recommend early onset of treatment in order to prevent an ischemic event [28]. Eastman et al. demonstrated a reduced stroke rate from 15.3 to 3.8% when time to diagnosis and thus start of treatment was lowered [45]. A recommendation of early treatment is in accordance with existing guidelines constituted from older primary studies than those included in our work [26, 27, 29, 31, 34, 72]. While earlier studies utilized systemic heparin infusion, more recent work has examined the use of oral antiplatelet or LMWH [7, 19, 43]. No randomized study has been performed comparing antiplatelet to anticoagulation in BCVI patients. Various publications show that several different treatment plans have been deployed often at the discretion of the attending physicians and that no drug has proven more effective than others [19, 73, 74]. There is no evidence that double platelet inihibitors or a combination of drugs is more effective than a single-drug regimen [75].

In a randomized study on spontaneous dissections of vertebral and carotid arteries it was found an equivalence of treatment result between antiplatelet and anticoagulation regimens [76]. As systemic heparinization is more labor-demanding than LMWH through the need of monitoring (aPTT or antifactor Xa heparin assay) we recommend the use of LMWH for the initial treatment. Systemic heparinization has previously been associated with increased risk of hemorrhage in the trauma population [77]. LMWH has a relatively short half-life of approximately 12 h and may be partially reversed by the use of protamine sulphate in case of hemorrhagic complications or pending surgical treatment [78]. LMWH may be continued throughout the entire treatment period, but oral antiplatelets should be used if feasible. We recommend low dose ASA (75 mg × 1) as this oral antiplatelet is readily available, cheap, easily administered even in children and as effective as a higher dose ASA in stroke prevention [50, 68, 79].

A strong recommendations as to exact length of treatment cannot be made. In a retrospective study on 29 patients, Rao et al. found that mean time to luminal recovery in patients with spontaneous and traumatic carotid dissections was 11.2 months [80]. Others have suggested that cervical vessel injuries that resolve do so within 3–6 months indicating that this may be sufficient for most patients [76, 81]. There is a paucity of data and most studies with long-term follow-up are on patients with spontaneous dissections. Luminal stenosis from atherosclerotic plaques represent a clinically different setting than that seen in trauma patients with persistent vessel narrowing.Whether a remaining vessel narrowing after 3–6 months in an asymptomatic trauma patient represent an indication for continued medical treatment or endovascular intervention is undetermined.

Endovascular treatment of BCVI has evolved over the past 20 years but remains controversial as complication rates, stent patency and stroke rates vary [30, 74, 82–84]. A pseudoaneurysm represents a vascular area with reduced flow and potential clot formation. Patients with this type of BCVI have often been considered candidates for endovascular intervention as the aneurysms rarely disappear and may produce cerebral emboli [69, 85]. Grade IV injuries (vessel occlusion) have also been suggested as indication for intervention in order to avoid recanalization and embolic events [86]. However, the need for double platelet treatment after stent placement is problematic in trauma patients. The current literature is divergent and inconclusive thus making clinical recommendations difficult. Each institution should consider its own experience with this technique and tailor any endovascular procedures in BCVI patients accordingly.

### Clinical question 4: How should patients with vessel injury be handled over time?

#### Recommendation

A follow-up CTA after approximately 7 days and 3 months is recommended. Strength of recommendation: Conditional.

Level of evidence: Low.

#### Evidence and rationale

As CTA may display false-positive findings we recommend a repeat scan after approximately 7 days. This may confirm the diagnosis and strengthen the indication for continued treatment or rule out the diagnosis in cases where the initial CTA was misinterpreted or displayed vessel spasm [20]. In the latter situations treatment may be halted. This recommendation is in accordance with previous guidelines [27, 72].

Follow-up studies on BCVI patients are few but seem to indicate that healing of the vessel lesion is reversely associated with injury grade: The higher Biffl injury grade (III and IV) lesions are less likely to improve than the low grades [69]. In a study of 110 patients with blunt carotid injuries, angiographic follow-up at a mean of 6 months was available in 50 patients demonstrating stable or improved findings in 75% of cases [74]. Franz et al. re-imaged 17 of 29 patients with BCVI with complete resolution in 84% at a mean of 9.2 weeks [87]. A final CTA after 3 months may thus serve as a guide for the clinician in deciding whether to continue or end antithrombotic treatment.

## Summary

The current guideline recommends using the expanded Denver screening criteria and CTA for the detection of BCVI in the in-hospital trauma population. Early antithrombotic treatment should be commenced as soon as considered safe and continued for at least 3 months when BCVI is detected. A follow-up CTA after approximately 7 days is recommended in order to confirm or reject the diagnosis. A final imaging at 3 months may serve as guidance for further individual treatment. There is an obvious need for more studies providing better data regarding incidence, yield of screening criteria, stroke rates and long term results. We encourage other institutions to address these issues and suggest utilizing results from trauma databases or through prospective studies.

## Additional files

Additional file 1: Overview of MesH terms used in the systematic searches. (DOC 41 kb)

Additional file 2: Overview of studies included after systematic searches and evaluation. (XLS 55 kb)

## Abbreviations

AGREE II: Appraisal of guidelines for research and evaluation
ASA: Acetyl salicylic acid
BCVI: Blunt cerebrovascular injury
CTA: Computed tomography angiography
DSA: Digital subtraction angiography
GRADE: Grading of recommendations, assessment, development and evaluations
LMWH: Low-molecular weight heparin
