# Supplementary material for: Best practice guidelines for blunt cerebrovascular injury (BCVI)
Source: Scand J Trauma Resusc Emerg Med. 2018 Oct 29;26:90. doi: 10.1186/s13049-018-0559-1 (PMC6206718; doi:10.1186/s13049-018-0559-1)
Supplement: Supplementary file 1 — Overview of MesH terms used in the systematic searches. (DOC 41 kb) [file 13049_2018_559_MOESM1_ESM.doc]

Additional file 1

Appendix 1 . Overview of MesH terms used in the systematic searches

| 1. vertebral artery/ |  |
| --- | --- |
| 2. vertebral artery/ and artery injury/ |  |
| 3. carotid artery injury/ |  |
| 4. exp carotid artery/ |  |
| 5. exp carotid artery/ and artery injury/ |  |
| 6. artery dissection/ |  |
| 7. 1 or 4 |  |
| 8. 6 and 7 |  |
| 9. 2 or 3 or 5 or 8 |  |
| 10. ((carotid or vertebral) adj arter* adj (injur* or dissect* or trauma)).mp. [mp=title, abstract, heading word, drug trade name, original title, device manufacturer, drug manufacturer, device trade name, keyword] |  |
| 11. (carotid adj3 ((false adj aneurysm*) or pseudoaneurysm*)).ti,ab. |  |
| 12. ((blunt or traumatic) adj ((cerebro adj vascular) or cerebrovasc* or cerebro-vascular) adj (injur* or dissect* or trauma)).mp. |  |
| 13. ((carot* or cerebr*) and (bcvi or tcvi)).mp. |  |
| 14. (carotid adj3 ((false adj aneurysm*) or pseudoaneurysm*)).mp. |  |
| 15. 10 or 11 or 12 or 13 or 14 |  |
| 16. limit 15 to yr="2015 -Current" |  |
| 17. 9 or 16 |  |
| 18. limit 17 to exclude medline journals |  |
| 19. 9 or 15 |  |
| 20. limit 19 to exclude medline journals |  |

| 1. Vertebral Artery/in [Injuries] |  |
| --- | --- |
| 2. Carotid Artery Injuries/cl, di, ep, et, mo, pa, ra, su, th, us [Classification, Diagnosis, Epidemiology, Etiology, Mortality, Pathology, Radiography, Surgery, Therapy, Ultrasonography] |  |
| 3. exp Carotid Arteries/in [Injuries] |  |
| 4. Vertebral Artery Dissection/cl, di, ep, et, mo, pa, ra, su, th, us [Classification, Diagnosis, Epidemiology, Etiology, Mortality, Pathology, Radiography, Surgery, Therapy, Ultrasonography] |  |
| 5. ((carotid or vertebral) adj arter* adj (injur* or dissect* or trauma)).mp. [mp=title, abstract, original title, name of substance word, subject heading word, keyword heading word, protocol supplementary concept word, rare disease supplementary concept word, unique identifier] |  |
| 6. (carotid adj3 ((false adj aneurysm*) or pseudoaneurysm*)).ti,ab. |  |
| 7. ((blunt or traumatic) adj ((cerebro adj vascular) or cerebrovasc* or cerebro-vascular) adj (injur* or dissect* or trauma)).mp. |  |
| 8. ((carot* or cerebr*) and (bcvi or tcvi)).mp. |  |
| 9. (carotid adj3 ((false adj aneurysm*) or pseudoaneurysm*)).mp. |  |
| 10. 5 or 6 or 7 or 8 or 9 |  |
| 11. limit 10 to yr="2015 -Current" |  |
| 12. 1 or 2 or 3 or 11 | |
